# Constructed wetland as a green remediation technology for the treatment of wastewater from underground coal gasification process

**DOI:** 10.1371/journal.pone.0300485

**Published:** 2024-03-12

**Authors:** Łukasz Jałowiecki, Aleksandra Strugała-Wilczek, Katarzyna Ponikiewska, Jacek Borgulat, Grażyna Płaza, Krzysztof Stańczyk

**Affiliations:** 1 Environmental Microbiology Unit, Institute for Ecology of Industrial Areas, Katowice, Poland; 2 Department of Energy Saving and Air Protection, Central Mining Institute, Katowice, Poland; 3 Faculty of Organization and Management, Silesian University of Technology, Zabrze, Poland; Siksha O Anusandhan University Institute of Technical Education and Research, INDIA

## Abstract

The wastewater from underground coal gasification (UCG) process has extremely complex composition and high concentrations of toxic and refractory compounds including phenolics, aliphatic and aromatic hydrocarbons, ammonia, cyanides, hazardous metals and metalloids. So, the development of biological processes for treating UCG wastewater poses a serious challenge in the sustainable coal industry. The aim of the study was to develop an innovative and efficient wetland construction technology suitable for a treatment of UCG wastewater using available and low-cost media. During the bioremediation process the toxicity of the raw wastewater decreased significantly between 74%—99%. The toxicity units (TU) ranged from values corresponding to very high acute toxic for raw wastewater to non-toxic for effluents from wetland columns after 60 days of the experiment. The toxicity results correlated with the decrease of some organic and inorganic compounds such as phenols, aromatic hydrocarbons, cyanides, metals and ammonia observed during the bioremediation process. The removal percentage of organic compounds like BTEX, PAHs and phenol was around 99% just after 14 days of treatment. A similar removal rate was indicated for cyanide and metals (Zn, Cr, Cd and Pb). Concluded, in order to effectively assess remediation technologies, it is desirable to consider combination of physicochemical parameters with ecotoxicity measurements. The present findings show that wetland remediation technology can be used to clean-up the heavily contaminated waters from the UCG process. Wetland technology as a nature-based solution has the potential to turn coal gasification wastewater into usable recycled water. It is economically and environmentally alternative treatment method.

## Introduction

Although the underground coal gasification (UCG) process generates a smaller amount of wastewater than traditional coal mining, it however the wastewater contains a high concentration of environmentally hazardous compounds [1, 2]. The release of numerous organic and inorganic contaminants from coal tars and ashes produced is connected with heterogeneous and homogenous reactions during different stages of UCG process such as pyrolysis, reduction, oxidation [3, 4]. Waste water stream from the UCG process is an important source of environmental pollution disrupts ecological sustainability and causes human health risk. It contains extremely complex high-concentration aromatic hazardous, toxic and refractory compounds including phenolics, polycyclic aromatic hydrocarbons (PAHs), nitrogen heterocyclic compounds (NHCs) and long chain n-alkanes., potentially hazardous and harmful to the environment [5–7].

Nowadays, UCG wastewater treatment has been considered as a emerging challenge to the sustainable development of coal industry. The extensive effort has been devoted to developing various cost-effective and environmentally friendly processes to removal of hazardous and refractory organic compunds from the wastewater. So far, integrated engineering systems including biological and chemical advanced treatment system as holistic approach have been considered to clean up of UCG wastewater [2, 8–10]. The development of an appropriate treatment method to remove pollutants from UCG wastewater is extremly important for the successful application of UCG technology in energy transformation.

As described in the literature, a lot of industrial wastewater has been treated by biological processes, among which the constructed wetlands (CWs) as nature-based treatment technology are good solutions. The proven advantages of CWs as green and sustainable technology has been discussed by many researchers and resulted in numerous applications [11–20]. Literature analysis showed that so far, CWs have not been applied to the treatment of wastewater from the coal industry.

In this context, the aim of this work was to develop an innovative and efficient wetland construction technology suitable for the treatment of wastewater from underground coal gasification process using available and low-cost media, which allowed to deepen the knowledge about working conditions and possible applications of CWs. In our study the ability of vertical flow constructed wetland to treat UCG wastewater was tested.

## Materials and methods

### UCG experiments and production of UCG post-processing wastewater

Three separate experimental simulations of UCG were carried out in a large-scale *ex-situ* installation located in the Barbara Experimental Mine in Mikołów, a part of the laboratory facilities of the Center for Clean Coal Technologies in GIG Research Institute (Katowice, Poland).

Two Polish coals from “Piast-Ziemowit” mine and “Wesoła” mine were gasified. Before gasification, raw coals were subjected to analysis in the accredited laboratory in GIG Research Institute (Katowice, Poland). A short summary of the basic properties of coals is presented in Table 1. Both raw coals have a relatively high content of volatile matter (approx. 30%). “Piast-Ziemowit” coal sample has a higher content of moisture and ash in comparison to the “Wesoła” coal. Coal from the "Wesoła" mine contains about two times less moisture and a lower content of ash, is more coalified and has a greater calorific value. Due to their properties, both coals can be classified as medium-rank bituminous coals [21, 22]. The raw coals were subjected to gasification using oxygen-enriched air (OEA) as a gasifying agent (Experiment 1 and Experiment 3). Additionally, the “Piast-Ziemowit” coal was gasified in the oxygen atmosphere (Experiment 2). Characteristics of the UCG experiments are presented in Table 2. Detailed information on the UCG simulations was described by Wiatowski et al. [21].

**Table 1:** Comparison of some parameters of coals used in the experiments.

Parameters (units)	Coal	
	“Piast-Ziemowit”	“Wesoła”
Moisture W^a^ (%)	7.47	3.49
Ash A^a^ (%)	7.64	2.15
Volatile matter content V^a^ (%)	30.49	30.12
Lower heating value Q^a^ (kJ kg^-1^)	26,103	31,543
Total sulphur S_t_^a^ (%)	0.99	0.21
Carbon C^a^ (%)	68.62	82.01
Hydrogen H^a^ (%)	4.30	5.18
Nitrogen N^a^ (%)	1.08	2.24
Oxygen O^a^ (%)	10.20	4.83

^**a**^Analytical state

**Table 2:** General assumptions and characteristics of conducted UCG Experiments 1–3 (Wiatowski et al., [21]).

Parameters (units)	Experiment no. 1	Experiment no. 2	Experiment no. 3
Coal origin	"Piast-Ziemowit" mine (Poland)		"Wesoła" mine (Poland)
Gasifying agent	OEA	Oxygen	OEA
Installation pressure	Ambient	Ambient	Ambient
Coal block dimensions (m)	0.6 x 0.8 x 2.5	0.5 x 0.7 x 2.0	0.5 x 0.7 x 2.0
Mass of coal inside the reactor (kg)	1225	687	830
Experiment duration (h)	56	72	72
Amount of coal gasified (kg)	140.9	323.9	165.3
Wastewater produced* (kg)	234 (1018)	364 (1372)	189 (1197)
Wastewater production rate (kg/h)	4.18	5.01	2.63
Wastewater outflow (kg/kg gasified coal)	1.66	1.12	1.14

* the real quantity of post-process water obtained from coal gasification after subtracting the volume of the water added to the scrubber; the total amount of water before correction is given in brackets.

### UCG wastewater sampling

Three post-process wastewater average samples were generated as a result of three different coal gasification experiments differing in the coal rank and gasifying agent. Wastewater collected after every completion of each UCG process were produced in the water scrubber and represents the average sample of wastewater for a given gasification experiment. The addition of water to the scrubber prevented clogging the process gas discharge pipes. The actual volume of wastewater generated in each of conducted UCG processes was obtained by subtracting the water that has been injected to the water scrubber during the experiment with the flow rate 14 kg h^-1^.

The total amount of raw wastewater generated as a result of coal gasification process was collected in a plastic tank with a capacity of 1 m^3^ (Mauser type) and after mixing and pouring into smaller containers, the samples were transported to the laboratory. Subsequently, wastewater samples were filtered through a 0.45μm pore diameter membrane filter under vacuum to remove coal tar and other undissolved residues before laboratory analysis. All raw wastewater samples were characterized by an unpleasant odour and a certain amount of fine suspension. The samples remained slightly coloured after filtration. All filtrates were stored at 4°C until analysis. The raw wastewater from each of the three UCG experiments was biologically treated with constructed wetland column set. The effluents from the constructed wetlands were collected after 14 and 60 days for physico-chemical analysis. The toxicity analysis of effluents was performed after 14, 30, 45, 60, 75 and 150 days.

### Wetland column set up

Laboratory-scale vertical subsurface flow constructed wetlands (VSF CWs) were set up in columns made of poly-methyl methacrylate polymer (PMMA). A total of four columns, one control and three experimental columns were set up. In experimental columns, the raw post-processing wastewater from the UCG processes was used.

The raw wastewater from UCG Experiment No. 1 was used in the constructed wetland marked No. 1 (CW1). Similarly, In the case of the raw wastewater from UCG experiments Nos. 2 and 3 was used to set up constructed wetlands Nos. 2 (CW2) and 3 (CW3).

**Fig 1:**
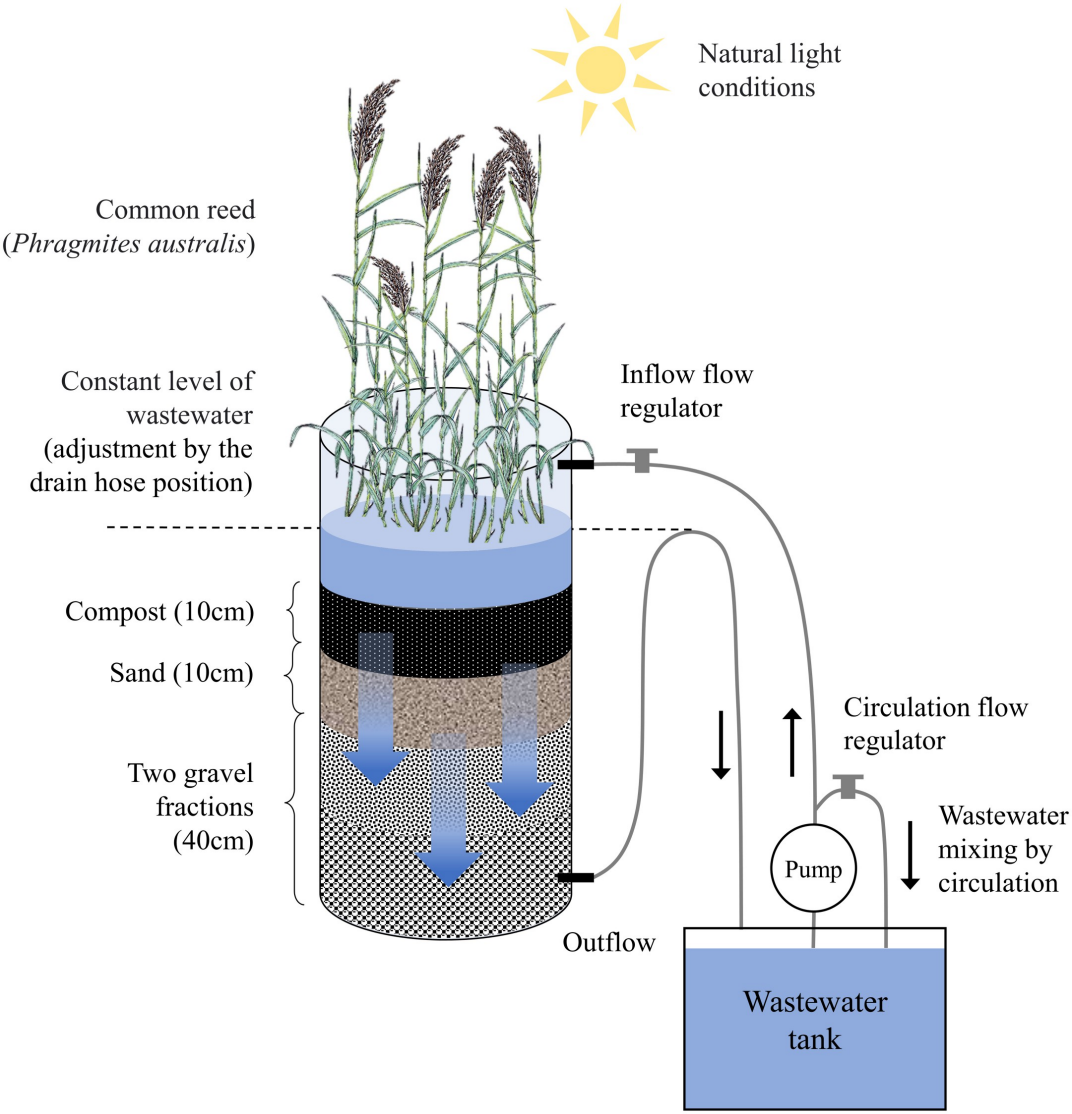
Scheme of the vertical subsurface flow constructed wetlands (VSF CWs) used in the bioremediation experiment.

The columns were filled with sand and gravel, a filling typically used in reed-bed wastewater treatment plants and the top layer of the column was green compost, whose task was to provide nutrients to the system. Eight cuttings of common reed (*Phragmites australis*) with a stem height of about 30 cm were planted in each column. The systems were stabilized with tap water for 2 weeks while maintaining the same flow parameters as when the systems were fed with UCG wastewater. The plants grew under natural light conditions. At the beginning of the experiment, the tanks placed next to a given experimental column contained 70 liters of wastewater. The raw wastewater passed through the column and returned to the tank by recirculation, where it was constantly mixed by the pump. Fig 1 shows an illustrative diagram of the experimental set-up. Technical details of the laboratory-scale constructed wetlands including column effluent retention time and water storage capacity of the bed are provided in Table 3.

**Table 3:** Technical and working parameters of laboratory scale vertical-subsurface flow constructed wetlands (VSF CWs).

Column parameters^a^		Values
Diameter		0.30 m
Height		0.80 m
Column area		633.5 cm^2^
Filling layers	(1) green compost	0.10 m
	(2) sand <1.0 mm	0.10 m
	(3) gravel fraction 5.0–8.0cm	0.20 m
	(4) gravel fraction 3.0–5.0cm	0.20 m
Volume		38 L
Maximum water volume		11.3 L
Water storage capacity of the bed		0.8 L
Average flow through the column (Q)^b^		5.65 L d^-1^
Hydraulic load		30 cm d^-1^
Hydraulic retention time (HRT)		2 day

^a^The columns were made of translucent Plexiglas. For this reason, each column was wrapped with aluminium foil from the outside to prevent algal blooms;

^b^Flow parameters were monitored at least once a day

### Physicochemical analysis

Optimally selected techniques and analytical methods used for the analysis of post-process UCG wastewater and effluents from the wetland columns are presented in detail in S1 Table. The analytical methods were selected appropriately according to the expected high content of impurities in the UCG polluted wastewater. The key instrumental equipment used for the analysis was IC analyzer Dionex ICS-5000 from Thermo Fisher Scientific (determination of inorganic anions); ICP-OES analyzer Optima 5300 DV from Perkin Elmer (determination of metals and metalloids); HS-GC-MS analyzer from Agilent Technologies (BTEX analysis); HPLC analyzer 1200 series with FLD detector from Agilent Technologies (PAHs analysis). All physico-chemical wastewater analysis were conducted in the accredited laboratories of GIG Research Institute in accordance with the currently applicable standards.

### Toxicity evaluation

Toxicity assessment of raw wastewater samples and effluents from wetland columns was conducted with the Microtox^®^ test recommended by ISO Standard 11348:1998. The test was carried out in the Microtox M500 toxicity analyzer according to the standard procedure [23, 24] with some modifications. In the test commercially available lyophilised bacteria *Vibrio fischeri* (NRRL-B 11177) was applied. The determination of the acute toxicity was performed by a two-step analysis. At first, in the initial screening test the toxicity was determined on non-diluted samples. In a second step, toxicity was assessed on sample solutions diluted with 2% NaCl to achieve more than a 50% effect over the initial screening The luminescence inhibition after 15 min was taken as the endpoint. The EC50 values (concentration causing 50% reduction in the bioluminescence of the bacteria) and the corresponding 95% confidence intervals were computed using the MicrotoxOmni^®^ Software. Then, the toxicity unit (TU) as 1/ EC50 was calculated. Finally, obtained results were ranked based on TU values into one of five classes described by Persoone et al.[25]. Each sample was run in triplicates assay. Toxicity tests were evaluated in the microbiological laboratory of the Institute for Ecology of Industrial Areas (Katowice, Poland).

### Statistical and computional analysis

In order to show the changes in the physico-chemical parameters of wastewater after the UCG processes and their effects on toxicity, the PCA analysis was carried out on the basis of a correlation matrix. The range of continuous output variables has been standardised so that each contributes equally to the analysis. All calculations were performed using Dell Statistica (data analysis software), version 13.

For selected post-process wastewater contaminants, the degree of wastewater treatment (η_14,60_, %) was calculated, defined as the ratio of the amount of pollutants retained as a result of wastewater treatment to the amount of pollutants contained in raw wastewater. The percent reduction was calculated after 14 and 60 days of contact of wastewater with constructed wetlands according to the equation:

$$\eta_{14,60}(\%)=\frac{c_{e}-c_{0}}{c_{0}}\cdot 100$$ (1)

where:

*c*_*0*_—the concentration or index of pollutants in the raw wastewater (mg L^-1^),

*c*_*e*_—the concentration or index of pollutants in the wastewater after 14 or 60 days of contact with constructed wetland (mg L^-1^).

## Results and discussion

### Characterization of UCG raw wastewater samples

The real quantities of post-process water obtained from coal gasification experiments were 234 kg, 364 kg and 189 kg in Experiment 1, Experiment 2 and Experiment 3, respectively (Table 2). The total amount of produced UCG wastewater was higher in the case of coal gasification under oxygen (experiment No. 2) (Table 2). A higher amount of coal gasified in the process with oxygen in comparison to oxygen-enriched air (OEA) results in relatively lower amount of water per unit mass of coal (Table 2). The wastewater production rate was higher in the case of gasification of „Piast-Ziemowit” coal (experiments No. 1 and No. 2). This can be connected with significantly higher moisture content (8.50% vs 3.73%, respectively), and together with unreacted steam, water evaporation or hydrogen combustion may result in the relatively high wastewater outflow in the Experiment No. 1 (with OEA as the gasifying agent) in reference to the coal consumption.

Table 4 presents changes in physicochemical parameters in UCG wastewater during the treatment by the constructed wetlands after 14 days and 60 days. In accordance with the Best Available Techniques (BAT)recommendations for coke wastewater, which are similar to UCG wastewater in terms of composition, the pollutant concentrations in coking wastewater after treatment and before discharging it to water or land, should not exceed the following levels for selected parameters: COD < 220 mg L^-1^, BOD_5_ < 20 mg L^-1^, free sulfides < 0.1 mg L^-1^; thiocyanides < 4 mg L^-1^; free cyanides < 0.1 mg L^-1^, polycyclic aromatic hydrocarbons (PAHs) < 0.05 mg L^-1^, phenols < 0.5 mg L^-1^, total nitrogen (sum of ammonia nitrogen, nitrate nitrogen, and nitrite nitrogen) < 15–50 mg L^-1^ [25].

**Table 4:** The changes of the parameters of wastewater during the bioremediation process (mean values, n = 3). Parameters that are exceeded in relation to the normative values given in BAT document (2012) are marked in bold.

Parameters (units)	CW1			CW2			CW3		
	day 0^a)^	day 14	day 60	day 0^a)^	day 14	day 60	day 0^a)^	day 14	day 60
pH	2.6	5.2	6.3	1.8	5.1	6.4	7.0	7.7	7.1
Conductivity (mS cm^-1^)	2610	1860	2220	8640	2830	3300	1530	1480	1850
Redox (mV)	263	186	274	382	175	259	112	211	254
Ammonia NH_4_ (mg L^-1^)	180	140	42	160	110	98	190	130	<0.02
N-NO_3_ (mg L^-1^)	0.11	<0.10	36	0.12	<0.10	<0.10	<0.1	<0.1	58
N-NO_2_ (mg L^-1^)	<0.006	0.0079	11	<0.002	<0.002	0.011	0.016	0.033	0.21
Total nitrogen (mg L^-1^)	**150**	**120**	**83**	**120**	**90**	**82**	**150**	**100**	**63**
BOD (mgO_2_ L^-1^)	**360**	**260**	7	**550**	**320**	9	**170**	14	**28**
COD (mgO_2_ L^-1^)	**1130**	**660**	120	**1060**	**660**	72	**397**	143	129
TOC (mgC L^-1^)	330	220	44	350	210	26	130	53	46
Chlorides (mg L^-1^)	516	450	483	1120	961	1080	269	228	312
Sulfates (mg L^-1^)	154	216	276	106	78.8	<10	130	198	276
Nitrates (mg L^-1^)	0.50	<0.50	160	0.53	<0.50	<0.50	<0.5	<0.5	260
Nitrites (mg L^-1^)	<0.006	0.026	35	<0.006	<0.006	0.036	0.051	0.11	0.70
Total cyanides (mg L^-1^)	6.7	2.8	0.065	42	0.16	<0.002	5.1	0.064	0.070
Phenol index (mg L^-1^)	**94**	**60**	0.017	**165**	**68**	0.0091	**41**	0.014	0.0042
Total phosphorus (mg L^-1^)	0.12	0.24	0.29	0.10	0.22	<0.065	<0.065	2.43	0.57
Sulfides (mg L^-1^)	<0.02	0.023	<0.02	**0.25**	0.054	<0.02	<0.02	<0.02	<0.02
Fe (mg L^-1^)	3.64	2.42	0.11	22.7	29.8	0.22	0.12	1.22	0.15
Mn (mg L^-1^)	0.14	1.27	1.45	0.39	2.41	1.77	0.053	0.23	0.22
Sb (mg L^-1^)	0.24	0.16	0.10	<0.05	<0.05	<0.05	0.2	<0.05	<0.05
As (mg L^-1^)	<0.05	<0.05	<0.05	<0.05	<0.05	<0.05	<0.05	<0.05	<0.05
B (mg L^-1^)	0.28	0.70	0.48	1.75	1.77	1.49	0.26	0.30	0.44
Cr (mg L^-1^)	0.28	0.077	<0.005	4.83	0.12	0.0058	<0.005	<0.005	<0.005
Zn (mg L^-1^)	13.4	4.2	0.40	4.65	1.19	<0.05	0.92	0.11	<0.05
Al (mg L^-1^)	1.6	0.81	<0.10	3.02	2.58	0.17	0.13	<0.1	<0.1
Ca (mg L^-1^)	2.12	93.6	294	1.34	319	397	0.86	63.6	286
Cd (mg L^-1^)	0.0078	0.003	<0.001	0.0084	<0.001	<0.001	<0.001	<0.001	<0.001
Co (mg L^-1^)	0.0059	0.066	0.048	0.071	0.11	0.0052	<0.005	0.032	0.033
Cu (mg L^-1^)	0.013	0.099	<0.01	<0.01	<0.05	<0.01	0.026	<0.01	0.011
Mg (mg L^-1^)	0.45	8.19	18.3	0.39	21.0	25.6	0.16	7.01	22.1
Mo (mg L^-1^)	0.0063	<0.05	<0.005	<0.005	<0.05	<0.005	0.011	0.014	0.0065
Ni (mg L^-1^)	0.40	0.22	0.034	2.21	0.58	0.044	0.32	0.10	0.084
Pb (mg L^-1^)	1.25	0.31	<0.02	3.06	<0.05	<0.02	<0.02	<0.02	<0.02
Hg (mg L^-1^)	<0.0005	<0.001	<0.001	<0.001	<0.001	<0.001	<0.001	<0.001	<0.001
Se (mg L^-1^)	<0.05	<0.05	<0.05	<0.05	<0.05	<0.05	<0.05	<0.05	<0.05
Ti (mg L^-1^)	0.023	0.021	<0.005	0.069	0.005	0.005	<0.005	0.0071	<0.005
PAHs (μg L^-1^)	**188.26**	45	0.68	**1731**	9.5	5.5	**2057**	12	0.214
BTEX (μg L^-1^)	1357	745	<0.8	732	194	<0.4	604	5.32	1.40

^a)^ Raw UCG wastewater used in a bioremediation process with constructed wetlands (CW)

In analyzed samples, pH ranged from 1.8 to 7.0 (Table 4). The wastewater originated from the gasification of “Piast-Ziemowit” coal were strongly acidic, which may be related to the relatively high sulfur content in the raw coal (five times more compared to "Wesoła" coal). A significant increase in the pH of the raw water sample after experiments 1 and 2 (from 2.6 to 6.3 and from 1.8 to 6.4, respectively for raw wastewater 1 and raw wastewater 2) may be related to the leaching of potential impurities in the form of calcium and magnesium carbonates present in the quartz gravel filling the column. On the basis of a blank test, it was verified that in the case of contact of gravel with a neutral solution, the pH does not change (the blank test was carried out on tap water; the initial pH of the solution was 6.7, after 14 days it was 6.8, and after 30 days it slightly increased to 6.9). In the acid blank, the starting pH was 2.4 and increased to 5.9 after only 5 hours, then slowly increased to 6.4 (after 14 days) and 6.5 (after 30 days). Additional analysis carried out confirmed the increase in Ca and Mg concentrations in the tested samples over time (Table 4).

**Fig 2:**
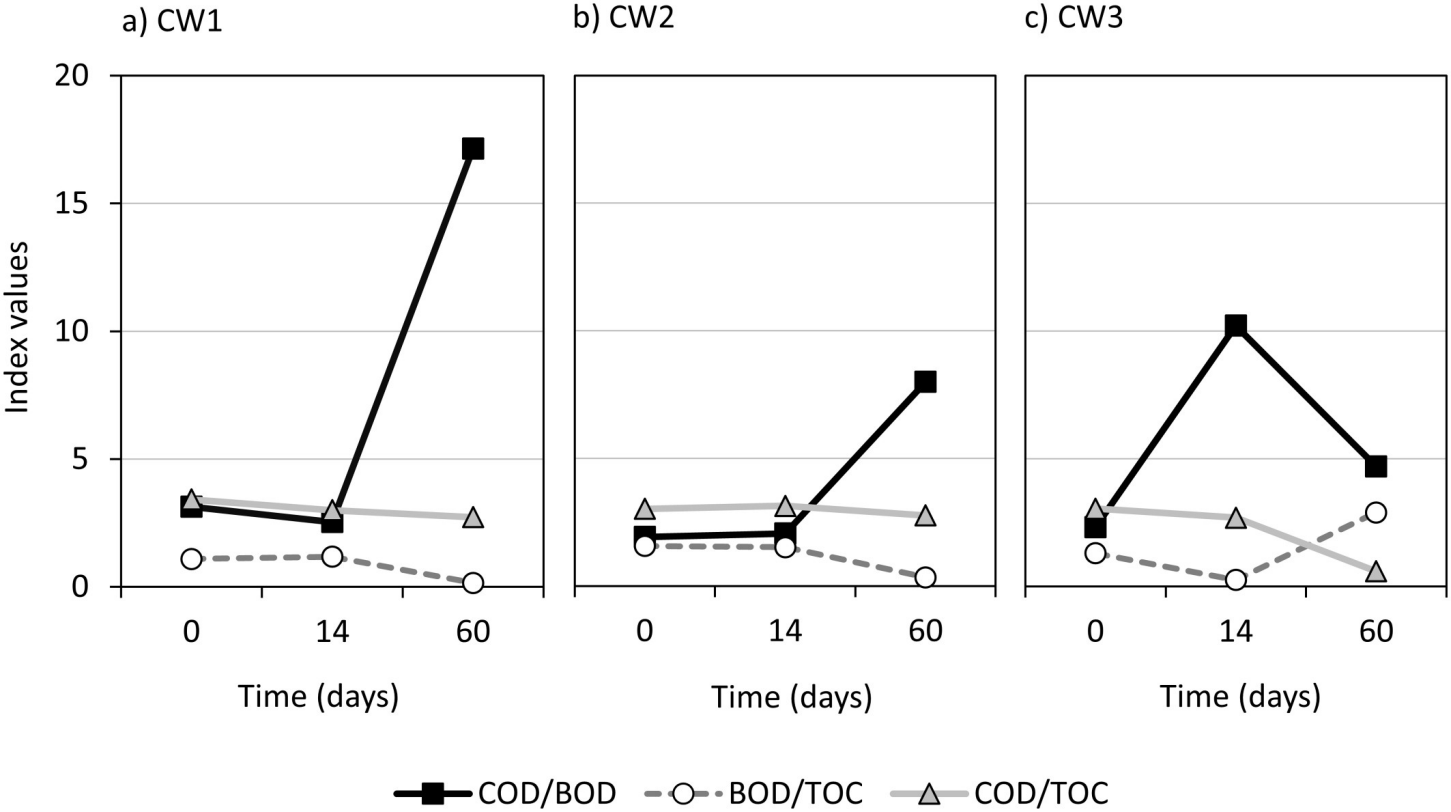
Change in the quotients of organic pollutant indicators in wastewater samples during bioremediation processes using CWs.

The raw wastewater after Experiment No. 2 had high conductivity. Total nitrogen consisted mainly of ammonia and remained constant in all three wastewater samples. Total cyanide content was found in all samples, with the highest concentration 42 mg L^-1^ determined in the raw wastewater 2, which deserves special attention due to the strongly acidic nature of the sample. The raw wastewater 1 and 2 contained significantly more COD, BOD, TOC and phenol index than the sample number 3 with moderate content of organic substances. Significant differences between the wastewater from coal gasification “Piast-Ziemowit” and “Wesoła” were also present in the TOC content (330–350 mg L^-1^ and 130 mg L^-1^, respectively). These differences may indicate a significant impact of the type and composition of the gasified coal on the composition of wastewater generated in the process, especially the concentrations of organic parameters. The relationship between the values of general organic pollutant indicators (such as BOD, COD, TOC) determines the susceptibility of wastewater to biological treatment plant processes (Fig 2) and is only an estimate, however can be important in terms of research on effective wastewater treatment using constructed wetlands.

The most significant relationship is attributed to the COD/BOD ratio, which can be an indicator of the degradation of organic matter and is constant for a specific type of wastewater. It is assumed that organic pollutants present in wastewater are biodegradable if this ratio is in the range of 1.5 to 2.5 [26, 27]. Above the value of 2.5, the decomposition of pollutants is slow and/or the wastewater contains organic substances that are hardly or not biodegradable, which is common in industrial wastewater. Among the tested UCG wastewater, the highest COD/BOD ratio occur for the wastewaters 1 and 2 after 60-days and for the wastewater 3 after 14-days of bioremediation (17.1, 8.0 and 10.2, respectively). The BOD/TOC ratio in the tested samples was below the typical range for raw wastewater (1.4–2.1) [26] confirming the content of hardly degradable pollutants. The COD/TOC quotient showed little variation and ranged from 2.70 to 3.42. According to Lee and Nikraz [27], the characteristic range of 2.5/3.0–4.0 is adopted for raw domestic wastewater. The presence of Hg was not observed in any sample, which is probably related to its high volatility and high UCG temperature. Process temperature for the experiment No. 2 with pure oxygen ranged from 1200°C to 1500°C and was higher than for the tests with OEA (the highest temperatures for experiments No. 1 and 3 were up to 1200°C). Toxic metals (Cd, Pb, Cr) were detected in wastewater after both gasification experiments of “Piast-Ziemowit” coal. The concentrations of other metals and metalloids were relatively low for all wastewater. Differences in the determination limits obtained for some parameters (e.g. Hg and N-NO_2_) result from the presence of interfering substances and the need for test sample dilution.

The characteristic feature of wastewater from industrial processes was the high content of BTEX and polyaromatic hydrocarbons (PAHs). The highest efficiency of CWs in removing pollutants was observed in the case of organic compounds (phenol index, PAHs, BTEX), especially in the case of water purification from the “Wesoła” coal gasification process. After bioremediation with CWs, the content of cyanides and metals (Fe, Cr, Zn, Ni, Pb and Ti) also decreased. M-Ridha et al. [28] used pilot scale horizontal subsurface flow constructed wetlands (HSSF-CWs) to remove Cd^2+^, Cu^2+^, and Ni^2+^ ions from simulated wastewater. A high removal efficiency reaching up to 99.3%, 99.5%, and 86.3% for Cd, Cu and Ni, respectively was noted. The concentration of Al and Cd decreased in the case of water from coal gasification “Piast-Ziemowit”. The conductivity initially decreases and then increases. As a result of nitrification, the level of nitrates increases, and wastewater is characterized by an unfavorably low carbon to nitrogen ratio, with the appropriate amount of available organic carbon being a necessary factor for denitrification [28]. The biological decomposition of organic matter in wastewater from the UCG of the “Piast-Ziemowit” coal begins to slow down after 14 days of treatment on the wetland. The situation is different for sewage after gasification of coal “Wesoła”, in the case of which an increase in the decomposition rate of organic substances was observed only after 14 days (based on the COD/BOD ratio). Also, the constant presence of phosphorus in wastewater confirms the research conducted by Mohamed et al. [29, 30] who stated that CWs have poor long-term phosphorus removal.

**Fig 3:**
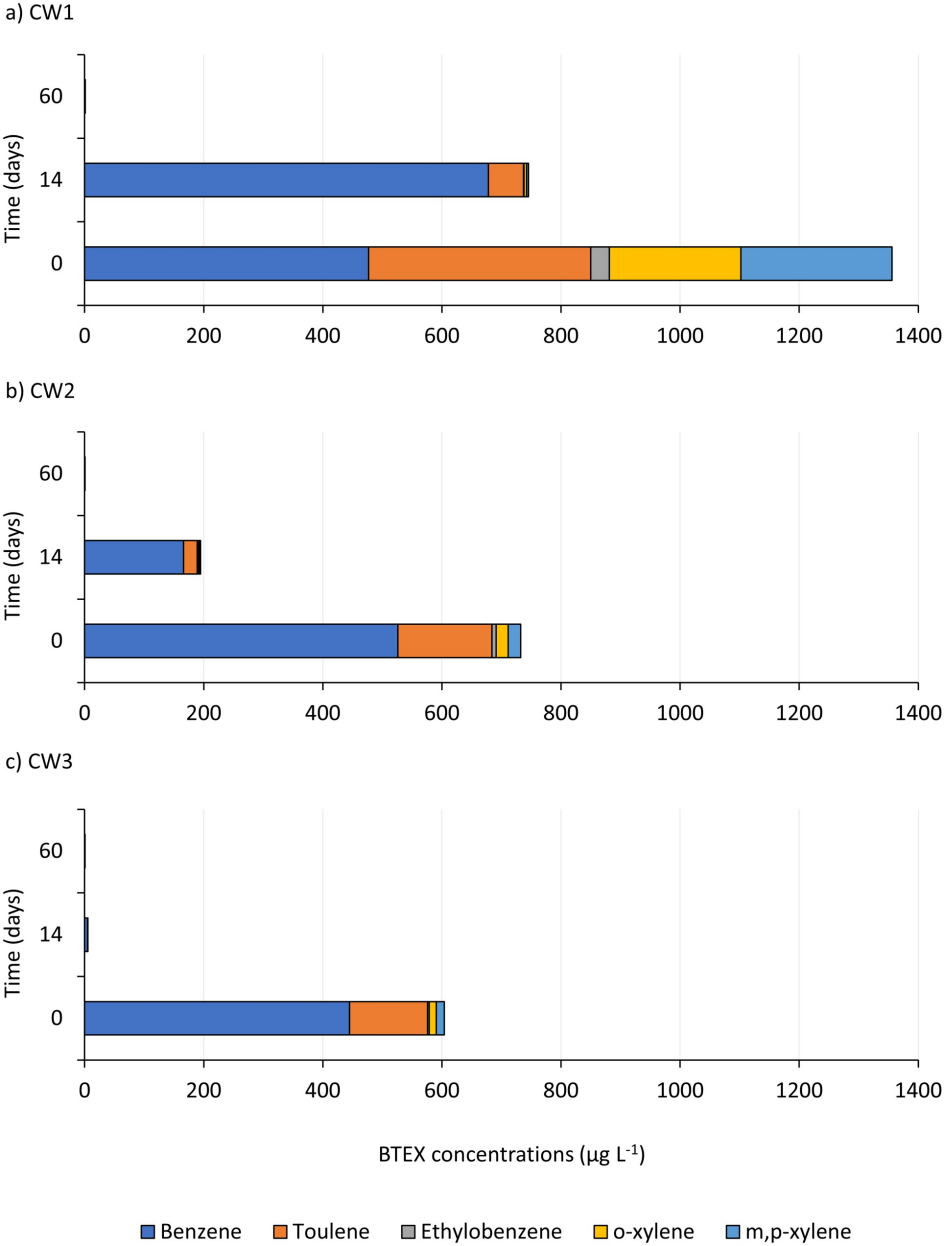
Changes in the individual BTEX compounds over the time of bioremediation.

In the case of BTEX, the largest share in each UCG wastewater sample had benzene (Fig 3), which is characterized by relatively high solubility in water (1.79 g L^-1^), however, on the examples of wastewater 1 and 2 it can be observed that an increase in temperature decreases the solubility of BTEX in water. The PAHs content in wastewater and the type of gasified coal or the gasifying agent. No relationship was found between the PAHs content in wastewater and the type of gasified coal or the gasification agent was observed.

**Fig 4:**
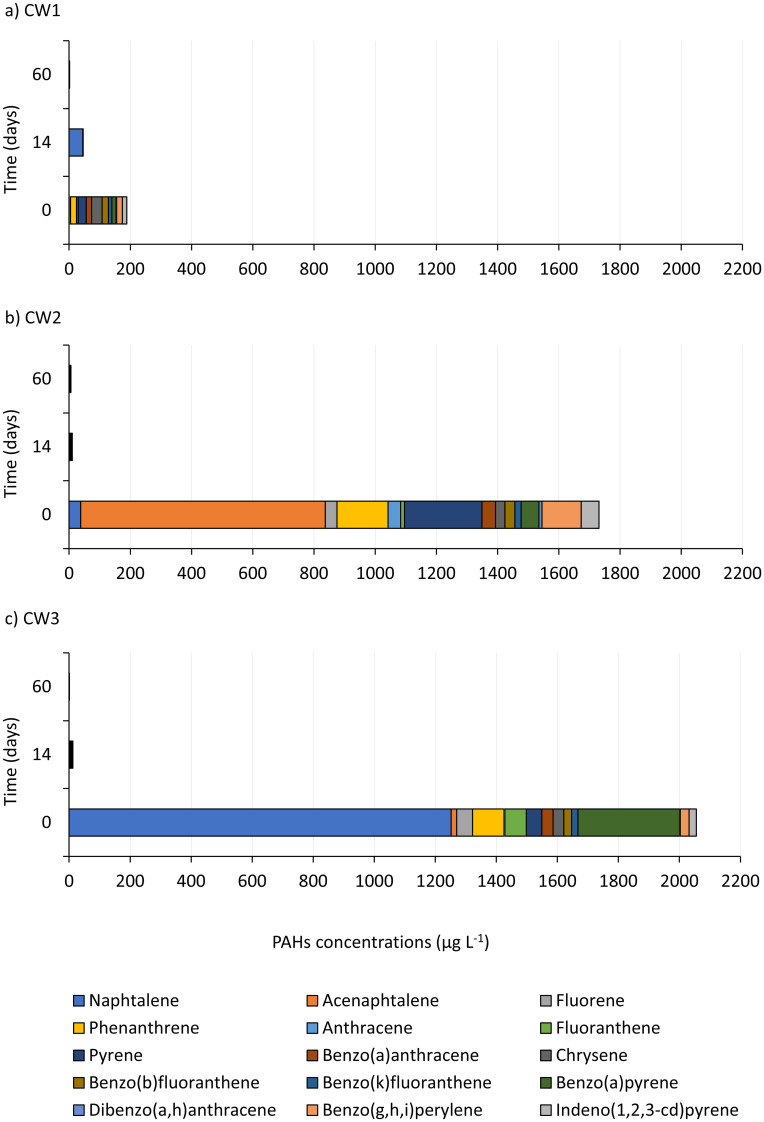
Changes in the individual PAHs compounds over the time of bioremediation.

The largest share of PAHs in the WW3 sample had naphthalene (Fig 4), and acenaphthene in the WW2 sample. In the WW1 sample with the lowest sum of PAHs, the shares of all individual components were at a similar level.

### Performance and treatment efficiency of the constructed wetland system

The degree of treatment, also known as the percentage of reduction, characterizes the efficiency of wastewater treatment plants and is standardized for key pollutants, such as BOD, COD, nitrogen and phosphorus compounds, in national and international legal acts, for example in the EU Council Directives regulating the minimum degree of pollutant removal for treated municipal wastewater discharged into water or the ground like [31]. The degree of wastewater treatment after 14 and 60 days (η_14,60_) is presented in Table 5.

**Table 5:** The degree (η_14_ / η_60_) of wastewater treatment from UCG processes after 14 and 60 days of bioremediation using CWs (mean values). A negative sign indicates a decrease in the content of the substance or measured parameter in question compared to the initial value measured at the beginning of the experiment. The parameters that were not detected in the tested samples and for which it was impossible to calculate the degree of wastewater treatment are marked with gray cells and the highest values above 80% are marked in bold.

Parameter	CW1		CW2		CW3	
	η_14_ (%)	η_60_ (%)	η_14_ (%)	η_60_ (%)	η_14_ (%)	η_60_ (%)
NH_4_	-22.2	-76.7	-31.3	-38.8	-31.6	-1**00.0**
N-NO_3_	-9.1	>32,000	-16.7	-16.7	0.0	>57,000
N-NO_2_	31.7	>183,000	0.0	450.0	106.3	1212
Total nitrogen	-20.0	-44.7	-25.0	-31.7	-33.3	-58.0
BOD	-27.8	**-98.1**	-41.8	**-98.4**	**-91.8**	**-83.5**
COD	-41.6	**-89.4**	-37.7	**-93.2**	-64.0	-67.5
TOC	-33.3	**-86.7**	-40.0	**-92.6**	-59.2	-64.6
Chloride	-12.8	-6.4	-14.2	-3.6	-15.2	16.0
Sulfate	40.3	79.2	-25.7	**-90.6**	52.3	112.0
Nitrate	0.0	>31,000	-5.7	-5.7	0.0	>51,000
Nitrite	333.3	>583,000	0.0	500.0	115.7	1272
Total cyanide	-58.2	**-99.0**	**-99.6**	**-100.0**	**-98.7**	**-98.6**
Phenol index	-36.2	**-100.0**	-58.8	**-100.0**	**-100.0**	**-100.0**
Total phosphorus	100.0	141.7	120.0	-35.0	>3,000	776.9
Sulfide	15.1	0.0	78.4	**92.0**		
Fe	-33.5	**-97.0**	31.3	**-99.0**	916.7	25.0
Mn	807.1	935.7	517.9	353.8	334.0	315.1
Sb	-33.3	-58.3			**-75.0**	**-75.0**
As						
B	150.0	71.4	1.1	-14.9	15.4	69.2
Cr	-72.5	**-98.2**	**-97.5**	**-99.9**		
Zn	-68.7	**-97.0**	-74.4	**-98.9**	**-88.0**	**-94.6**
Al	-49.4	**-93.8**	-14.6	**-94.4**	-23.1	-23.1
Cd	-61.5	**-87.2**	**-88.1**	**-88.1**		
Co	>1.000	713.6	54.9	**-92.7**	540.0	560.0
Cu	661.5	-23.1	400.0	0.0	-61.5	-57.7
Mo	693.7	-20.6	900.0	0.0	27.3	-40.9
Ni	-45.0	**-91.5**	-73.8	**-98.0**	-68.8	-73.7
Pb	-75.2	**-98.4**	**-98.4**	**-99.3**		
Hg						
Se						
Ti	-8.7	-78.3	**-92.8**	**-92.8**	42.0	0.0
PAHs	-76.1	**-99.6**	**-99.5**	**-99.7**	**-99.4**	**-100.0**
BTEX	-45.1	**-99.9**	-73.5	**-99.9**	**-99.1**	**-99.8**

After contact with CWs, the content of sulfate, nitrate, nitrite (nitrites are oxidized to nitrates and TOC decreases), total phosphorus, and some trace elements (Mn, B, Co, Cu and Mo) significantly increased in the tested wastewater. CWs showed the greatest effectiveness in the removal of organic pollutants such as phenol index, PAHs (higher treatment efficiency observed in the case of wastewater from the second coal gasification experiment “Piast-Ziemowit”), BTEX (better efficiency in wastewater after coal gasification “Wesoła”). The conductivity in wastewater initially decreases, but after 14 days it starts to increase. In wastewater after contact with CWs, definitely smaller amounts of cyanides and some metals were determined: Fe, Cr, Zn, Al only in the case of gasification of coal “Piast-Ziemowit”, Cd (also for coal “Piast-Ziemowit”), and Ni (lower removal efficiency in the case of sewage from coal gasification “Wesoła”), as well as Pb and Ti.

CWs were initially used to treat municipal wastewater containing high concentrations of nutrients [32, 33]. However, in recent years the evolution of CWs from simple to more advanced systems has evolved and various hybrid CWs have been applied to treat industrial wastewater from dairy and meat processing, the pulp and paper factory, brewery, tannery and olive mills [16, 17, 34, 35]. The number of CWs applications is constantly growing and includes several kinds of industrial wastewater, including petrochemical, acid mine wastewater and landfill leachates [12, 14, 33, 36–40].

In our study vertical flow constructed wetland to treat UCG wastewater was used to clean up the UCG post-processing wastewater. The research carried out in this article allowed to add another type of sewage to the above list. A high pollutant removal rate was obtained in the described wetland system. The removal percentage of organic compounds like BTEX, PAHs and phenol were 99% after 14 days of the treatment. A similar removal rate was indicated for cyanide and metals (Zn, Cr, Cd, Pb). The higher pollutant removal rate from our study were consistent to the results obtained by Saeed et al. [13] and Saeed & Khan [41]. While, Waly et al. [20] reported that the removal efficiency of PAHs in secondary treatment of traditional wastewater treatment plants (WWTPs) was lower, and ranged between 45%–82%.

Considering the complex composition of pollutants and the presence of recalcitrant organics in UCG post-processing wastewater, the combination of different types of materials and compost used in CWs increased removal rates due to high activities of plant root-associated microbiota with the combination of physicochemical processes. Also, the appropriate structure and properly selected operation parameters of CWs system were essential for achieving satisfactory removal rates of organic and inorganic pollutants during the experiment. Obtained in the study results indicate the potential of CWs to treat polluted and poorly biodegradable UCG post-processing wastewater.

### Evaluation of toxicity during the bioremediation process

**Fig 5:**
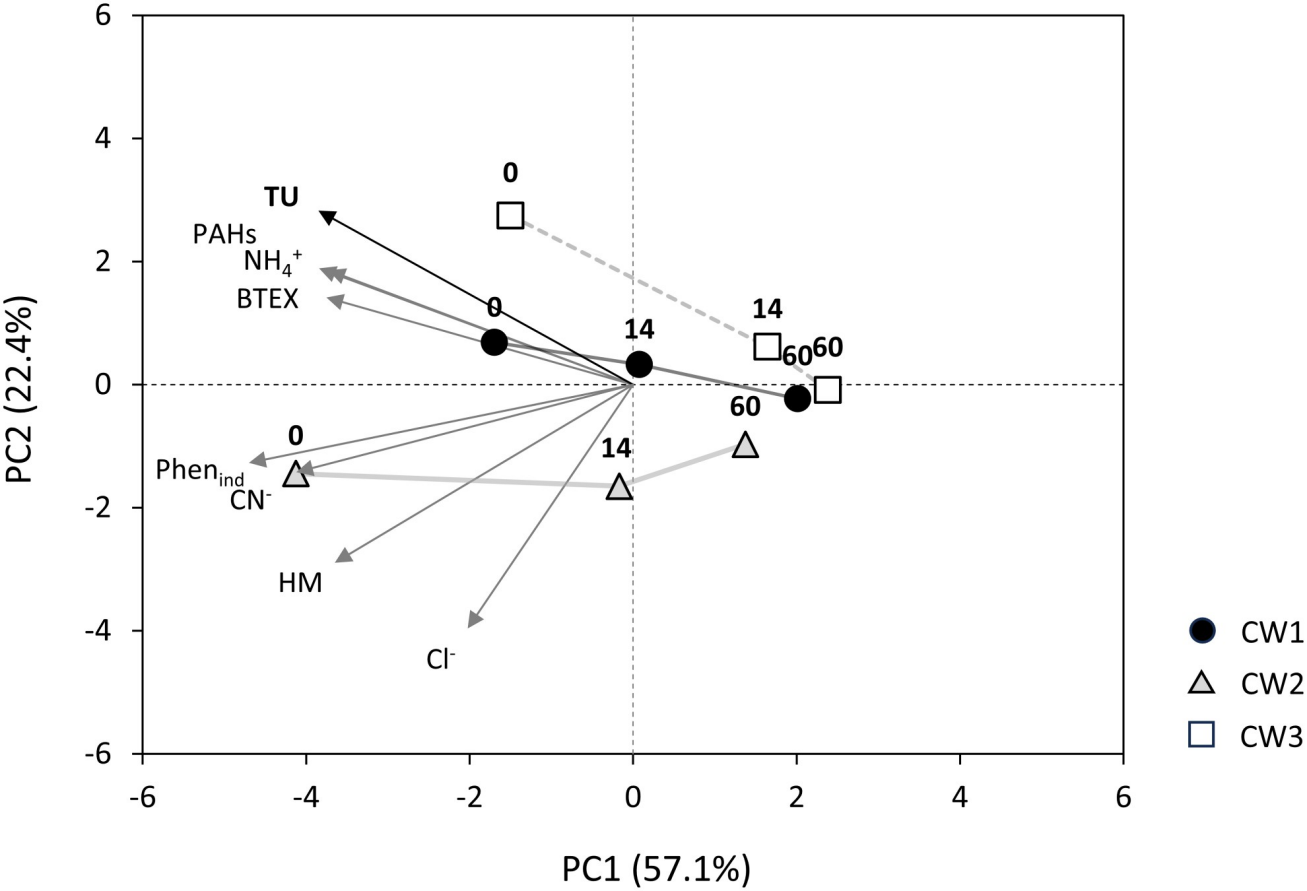
Dependence between selected physicochemical parameters and toxicity over bioremediation time evaluated by PCA analysis. Numbers above icons indicate day of measurement. Abbreviations: BTEX- volatile aromatic hydrocarbons, CN^-^-cyanides, HM- heavy metals including metalloids (As and Sb), PAHs- polycyclic aromatic hydrocarbons, Phen_ind_- phenol index, TU—toxicity unit.

The changes of toxicity as a function of bioremediation in wetland columns were determined over 150 days. The results are presented in Table 6. The toxicity indicator evaluated as Toxicity Unit (TU) was high for all three raw wastewater obtained from the UCG processes. The values of TU were 373, 405 and 732 for 1, 2 and 3 raw wastewater, respectively, and wastewater were classified to the fifth toxicity class (very high acute toxicity) according to classification proposed by Persoon et al. [25]. After 14 days of running the experiment, the toxicity of the wetland column effluents decreased significantly. The reduction of toxicity was 74% and 77% for raw wastewater 1 and 2, respectively, while, 99% reduction of toxicity was noted for raw wastewater 3. During the rest of the experiment, the toxicity of all wetland column effluents decreased, and at the end of the experiment the 1 and 3 effluent belonged to the first class of toxicity (no toxicity) with the TU values 0.9 and 0.7, respectively. After the end of the experiment, the effluent 2 belonged to the second class of toxicity (low toxicity) with the TU value of 1.1. Our results showed the toxicity of the wastewater samples ranged from very high acute toxicity (raw wastewater) to non-toxic (effluents from wetland columns) during the bioremediation process. The observed decrease was due to the conversion of the toxic compounds to less or non-toxic intermediates and by-products during experiment time. The obtained results correlated with some organic compounds such PAHs, BTEX, phenols and inorganic compound such as ammonia and cyanides (Fig 5). The mechanisms involved in pollutant removal in CWs is classified into biotic processes such as biodegradation, biofilm, root and plant uptake and physicochemical processes. Nevertheless, CWs are highly complex systems usually consisting of several layers such as compost, sand, gravel, soil that represent specific environments and microenvironments with different physicochemical and microbiological conditions involved in the removal of pollutants.

**Table 6:** Changes of effluents toxicity during the bioremediation experiment (mean ± SD, n = 3).

Time (days)	0	14	30	45	60	75	150
CW1							
EC50	0.00	1.02±0.015	0.83±0.028	27.98±3.51	36.91±2.60	33.60±1.25	86.00±2.12
TU	373.6±22.5	98.39±1.56	79.1±0.78	3.572±0.37	2.7±0.21	2.97±0.09	1.1±0.21
Toxicity class	V	IV	IV	III	III	III	II
CW2							
EC50	0.00	1.09±0.01	0.94±0.01	5.82±0.51	47.72±4.5	86.76±2.11	104±3.21
TU	405.4±8.76	91.74±0.56	89.7±0.28	17.19±1.73	2.09±0.22	1.15±0.25	0.9±0.03
Toxicity class	V	IV	IV	IV	III	II	I
CW3							
EC50	0.00	30.96±0.45	86.76±9.4	93±5.65	109±5.65	106±0.00	112±2.12
TU	732±15.67	3.2±0.05	1.15±0.15	1.28±0.07	0.8±0.07	1.0±0.00	0.7±0.07
Toxicity class	V	III	II	II	I	I	I

The evaluation of the remediation effect of natural and constructed wetland systems has been monitored by using only physicochemical parameters. However, several authors have emphasized the importance of including bioassays to monitor the effect of wetland remediation technologies and they introduced different toxicity tests to their research [42–47]. The Microtox test was found to be one of the most sensitive and to have a better ability to evaluate the toxicity of treated and nontreated wastewater samples [44]. In our study, the Microtox test has proven to be a useful complement to chemical analysis in terms of evaluating the course of wetland remediation technologies. As recommended by scientists, the ecotoxicity bioassays could be used as supplementary tools for monitoring the effectiveness of wetland remediation technologies.

CWs as nature-based processes remove pollutants from the water, avoiding the use of chemical products and the input of high amounts of external energy [33, 48, 49]. One of the positive characteristics of CWs is their work under controlled conditions such as the well-defined composition of media, plant types, flow patterns, controlled hydraulic pathways and retention time. Additional advantages of CWs include site selection, flexibility in sizing, and low operational and maintenance costs [32]. In the constructed wetlands the removal of pollutants is carried out by different physical, chemical, and biological processes like sedimentation, filtration, precipitation, ion exchange, adsorption, microbial processes for instance, nitrification, denitrification, sulphate reduction, carbon metabolization, and plant uptake. The activities of the processes are dependent on the interaction between plants, microorganisms, wastewater characteristics, media used and operation conditions [33, 50]. Traditionally CWs have been used for urban wastewater, stormwater runoff and domestic wastewater treatment, but in the last two decades, the applications for industrial wastewater purification increased due to the development of the technology and the extended research in this field.

## Conclusions

The vertical flow constructed wetlands with high activity of plant root-associated microbiota and a combination of physicochemical processes turned out to be a very good technology to clean up the post-processing wastewater from the underground gasification process. Also, the appropriate design and operating parameters of the CW system were essential for achieving a satisfactory pollutants removal rate during the experiment. Such results indicate the potential of CWs for the treatment of highly contaminated and poorly biodegradable industrial wastewater, such as UCG wastewater or coking wastewater. The conducted study allows to assume that despite the significant content of impurities, the UCG wastewater is suitable for biological treatment. The present findings show that wetland remediation technology can be used to clean-up the heavily contaminated waters from the UCG process. In environmental monitoring, in order to effectively assess remediation technologies, it is desirable to consider combining the use of traditional physicochemical parameters with ecotoxicity tests. To assess the toxicity of treated waters biological tests can be carried out together with physicochemical analyzes to ensure environmental safety and minimize ecotoxicological issues. Wetland technology has the potential to turn coal gasification wastewater into usable recycled water.

To understand the removal of pollutants by CWs, further research is needed, including balancing the process and examining the role of plants-microbes interactions in wastewater treatment.

## Supporting information

S1 Checklist Author formatting checklist. (DOCX)

S1 Table Methods used for the physicochemical analysis of UCG wastewater samples. (DOCX)
