# Supplementary material for: Constructed wetland as a green remediation technology for the treatment of wastewater from underground coal gasification process
Source: PLoS One. 2024 Mar 12;19(3):e0300485. doi: 10.1371/journal.pone.0300485 (PMC10931533; doi:10.1371/journal.pone.0300485)
Supplement: S1 Table — (DOCX) [file pone.0300485.s002.docx]

**Supplementary material**

Table S1. Methods used for the physicochemical analysis of UCG wastewater samples.

| Parameters | Method | Reference document |
| --- | --- | --- |
| pH | Potentiometric | PN-EN ISO 10523:2012 |
| Conductivity (mS/cm) | Conductometric | PN-EN 27888:1999 |
| Redox (mV) | Potentiometric (Ag/AgCl) | Laboratory own procedure |
| Ammonia NH_4_ (mg/L) | Flow injection analysis (FIA)  with spectrophotometric detection | PN-EN ISO 111732:2007 |
| Total nitrogen (mg/L) | High-temperature combustion  with chemiluminescent detection | PN-EN 12260:2004 |
| Biochemical oxygen demand (BOD) (mgO_2_/L) | Electrochemical | PN-EN 1899-2:2002 PN-EN ISO 5815-1:2019-12 |
| Chemical oxygen demand (COD) (mgO_2_/L) | Spectrophotometric | PN-ISO 15705:2005 |
| Total organic carbon (TOC) (mgC/L) | High-temperature combustion  with IR detection | PN-EN 1484:1999 |
| Chlorides (mg/L) | Titrimetric /  ion chromatography (IC) | PN-ISO 9297:1994 / PN-EN ISO 10304-1:2009 |
| Sulfates (mg/L) | Gravimetric /  ion chromatography (IC) | PN-ISO 9280:2002 / PN-EN ISO 10304-1:2009 |
| Nitrates (mg/L) | Spectrophotometric /  ion chromatography (IC) | Laboratory own procedure / PN-EN ISO 10304-1:2009 |
| Nitrites (mg/L) | Spectrophotometric /  ion chromatography (IC) | PN-EN 26777:1999 / PN-EN ISO 10304-1:2009 |
| Total cyanides (mg/L) | Continuous flow analysis (CFA) with spectrophotometric detection | PN-EN ISO 14403-2:2012 |
| Phenol index (mg/L) | Continuous flow analysis (CFA) with spectrophotometric detection | PN-EN ISO 14402:2004 |
| Sulfides (mg/L) | Flow injection analysis (FIA)  with spectrophotometric detection / spectrophotometric | Laboratory own procedure |
| Total phosphorus (mg/L) | Inductively coupled plasma - optical emission spectrometry (ICP-OES) | PN-EN ISO 11885:2009 |
| Fe, Mn, Sb, As, B, Cr, Zn, Al, Cd, Co, Cu, Mo, Ni, Pb, Se, Ti, Ca, Mg (mg/L) | Inductively coupled plasma - optical emission spectrometry (ICP-OES) | PN-EN ISO 11885:2009 |
| Hg (mg/L) | Cold-vapour atomic absorption spectrometry (CV-AAS) with the amalgamation technique | PN-EN ISO 12846:2012 +Ap1:2016-07;  US EPA 7473 |
| Polyaromatic hydrocarbons (PAHs):   - naphthalene - acenaphthene - fluorene - phenantrene - anthracene - fluoranthene - pyrene - benzo(a)anthracene - chryzene - benzo(b)fluoranthene - benzo(k)fluoranthene - bezo(a)pyrene - benzo(g,h,i)perylene - dibenzo(a,h)anthracene - indeno(1,2,3-cd)pyrene | High-performance liquid chromatography (HPLC-FLD) after pressure liquid-solid extraction (SPE) | PN-EN ISO 179993:2005; laboratory own procedure |
| Volatile aromatic hydrocarbons  (BTEX incl. styrene):   - benzene - toluene - ethylobenzene - o-xylene - m,p-xylene | Headspace analysis with gas chromatography and mass detection (HS-GC-MS) | PN-ISO 11423-1:2002 |
